# Laryngeal mask versus facemask in the respiratory management during catheter ablation

**DOI:** 10.1186/s12871-019-0924-2

**Published:** 2020-01-07

**Authors:** Takashi Koyama, Masanori Kobayashi, Tomohide Ichikawa, Yasushi Wakabayashi, Daiki Toma, Hidetoshi Abe

**Affiliations:** 1 Department of Cardiovascular Medicine, Matsumoto Kyoritsu Hospital, Habaue 9-26, Matsumoto, 390-8505 Japan; 2 Department of Gasteroenterological Surgery, Matsumoto Kyoritsu Hospital, Matsumoto, Japan

**Keywords:** Laryngeal mask, Catheter ablation, ETCO_2_, Capnography

## Abstract

**Background:**

The purpose of this study is to investigate if a laryngeal mask could improve respiratory condition during radiofrequency catheter ablation (RFCA).

**Methods:**

Twenty-four consecutive patients who underwent RFCA for atrial fibrillation were divided into two groups (Facemask group; *n* = 10, Laryngeal mask group; *n* = 14). All patients were completely sedated under intravenous anesthesia and fitted with artificial respirators during the RFCA. The capnography waveforms and their differential coefficients were analyzed to evaluate the changes of end-tidal CO_2_ (ETCO_2_) values, respiratory intervals, expiratory durations, and inspiratory durations.

**Results:**

During the RFCA, ETCO_2_ values of the laryngeal mask group were higher than those of the facemask group (36.0 vs. 29.2 mmHg, *p* = 0.005). The respiratory interval was significantly longer in the laryngeal mask group than those in the facemask group (4.28 s vs.5.25 s, *p* < 0.001). In both expiratory and inspiratory phases, the mean of the maximum and minimum values of CO_2_ was significantly higher when using a laryngeal mask than when using a facemask. The inspiratory-expiratory ratio of the laryngeal mask group was significantly larger than that of the facemask group (1.59 vs. 1.27, *p* < 0.001). The total procedure duration, fluoroscopic duration and the ablation energy were significantly lower in the laryngeal mask group than in the facemask group. The ETCO_2_ value is the most influential parameter on the fluoroscopic duration during the RFCA procedure (β = − 0.477, *p* = 0.029).

**Conclusions:**

The use of a laryngeal mask could stabilize respiration during intravenous anesthesia, which could improve the efficiency of RFCA.

## Background

Radiofrequency catheter ablation (RFCA) is an effective therapeutic option for the treatment of atrial fibrillation (AF) [1]. Additionally, with the advancement of technologies such as 3D mapping, the success rate and safety of AF catheter ablation continues to increase. Catheter ablation for AF is a relatively long surgery and to avoid pain due to cauterization, it is commonly performed under intravenous anesthesia [2]. When an RFCA is performed under sedation by intravenous anesthesia, breathing becomes unstable temporally and spatially due to obstruction of the upper airways. This situation causes problems such as: (1) decreased consistency between the geometry obtained from the 3D mapping and CT (2) decreased catheter static which causes difficult cauterization (3) drawing of air from the inserted sheath which increases the risk of air embolism [3]. To avoid such technical problems caused by respiratory instability associated with an RFCA, artificial respiration management is performed with a facemask [2] or a laryngeal mask. The invasiveness of respiration management using a laryngeal mask is midway between management using a facemask and that by tracheal intubation. When compared to tracheal intubation, the use of a laryngeal mask is lowly invasive, easy to operate, and has a low risk of pharyngeal and laryngeal injury [4, 5].

Capnography is used to continuously monitor ventilation to ensure that anesthesia is delivered safely [6]. Diseases and abnormalities related to breathing and circulation can be also be diagnosed quickly by analyzing capnography waveforms [7]. End-tidal CO_2_ (ETCO_2_) monitoring is extensively used as an objective parameter to determine whether appropriate ventilation is performed during operation.

There are only a few reports on the assessment and judgment of breathing conditions during an RFCA. Furthermore, the effects of the laryngeal mask in the respiratory management during RFCA have not been assessed extensively. Therefore, this study aimed to elucidate the differences between respiratory management using a laryngeal mask and a facemask and to demonstrate the beneficial effects of a laryngeal mask on the respiratory condition during an RFCA.

## Methods

### Subjects and study design

This study included 24 consecutive patients who underwent RFCA at our hospital for atrial fibrillation (paroxysmal, and persistent) from August 2018 to March 2019. We included patients who received pulmonary vein isolation without non-pulmonary vein (PV) ablation in this study. We excluded patients with left ventricular systolic function ≤50%, respiratory diseases such as chronic obstructive pulmonary disease, dialysis, and patients who did not require sedation (such as paroxysmal supraventricular tachycardia). Patients who received cryoablation were also excluded as it requires diaphragmatic pacing and makes an accurate assessment of respiratory movement temporarily difficult.

Blood samples were collected from the peripheral vessels of 24 enrolled patients before they underwent ablation procedures. Additionally, the subjects were screened for severe inflammation, heart failure, anemia and renal failure before the ablation procedure. The values of brain natriuretic peptide (BNP) and C-reactive proteins (CRP) were converted to logarithms. Renal function was measured based on the estimated glomerular filtration rate (eGFR). The patients were screened for sleep apnea using a standard digital pulse oximeter (PULSOX-300™, KONICA MINOLTA Inc.) before admission.

Two-dimensional, M-mode, and Doppler echocardiography (iE33; Philips Medical Systems, Andover, MA, USA) were performed to evaluate various parameters of heart functions in enrolled patients. The left ventricular ejection fraction was determined from an apical 4-chamber view using Simpson’s method. Left atirum (LA) diameter was measured in the parasternal long axis view from trailing edge of the posterior aortic root-anterior LA complex to the posterior LA wall at end-systole.

Intravenous anesthesia was used for all the subjects and RFCA was performed under complete sedation. Assisted respiration was performed using an artificial respirator (VELA Type D, Vyaire Medical Inc.). The attending physician decided whether to use a facemask or a laryngeal mask for airway management. The settings for artificial respiration were: SIMV mode, ventilation frequency = 10; pressure support = 6 cmH_2_O; and Positive End Expiratory Pressure (PEEP) = 5 cmH_2_O, FiO_2_ = 30–40%. Informed consent regarding the catheter ablation procedure and the use of data was provided for all patients. The design, protocol, and handling of patient data were reviewed and approved by the Matsumoto Kyoritsu Hospital ethics committee (approval No.2019–004).

### Intravenous sedation during the catheter ablation procedure

The patient was placed in a supine posture on the catheterization table. After confirming that there was nothing in the patient’s mouth (such as dentures), hydroxyzine pamoate (25 mg) was administered intravenously as premedication, and pentazocine (15 mg) was similarly administered as a sedative. After 5 min the patient’s vital signs and oxygen saturation levels were confirmed to be within normal limits. An Ambu-Bag (SPUR ll™, Ambu Inc. Denmark) and face mask (Disposable Face Mask, Vital signs Inc. USA.) with an oxygen flow rate of 10 L/min was gently placed on the patient’s nose and mouth followed by intravenous administration of propofol at 0.5 mg/kg/10 s until the patient fell asleep. After confirming that the olfactory hair reflex had disappeared, the patient’s head was placed in the Magill position, the mandible was lowered manually, and the mouth was opened. A medical lubricating gel was applied and a laryngeal mask (i-gel™: Intersurgical Ltd., UK) was slowly inserted. A laryngeal mask appropriate for the patient’s weight and physical constitution was selected and an Ambu-Bag was placed over the laryngeal mask. The bag was gently compressed to raise the chest, and after confirming that breathing sounds could be heard, the artificial respirator was attached. Insertion of the sheath and administration of dexmedetomidine hydrochloride was done simultaneously at 6 μg/kg/h by continuous intravenous drip infusion over 10 min (initial bolus). Next, according to the patient’s condition, a maintenance dose at 0.2–0.7 μg/kg/h (maintenance bolus) was administered to reach the optimum intravenous level. The olfactory hair reflex was confirmed every 20 min and if present, propofol was administered intravenously at 0.5 mg/kg/10 s until the reflex disappeared. During the RFCA, if the patient moved due to pain, propofol was again administered intravenously.

During the RFCA, the right internal jugular and right femoral veins were catheterized and a sheath was inserted in the femoral artery to monitor blood pressure. After a transseptal puncture using intracardiac echocardiography, a circular mapping catheter (Lasso, Biosense Webster Inc., Diamond Bar, CA, USA) was placed on the ostium of each PV atrium. The PV isolation was performed with a 3.5-mm tip, open-irrigated ablation catheter (THERMOCOOL™, Biosense Webster Inc. USA, or TACTICATH™ Abbot Inc. USA) to achieve electric isolation of the PV potential. All ablation procedures were performed with a 3D electroanatomical mapping system (CARTO™, Biosense Webster Inc. USA, or Ensite Navix™, Abbot Inc. USA). The RF energy output was titrated to 25–35 W at a flow rate of 17–30 ml/min, with a maximum temperature of 42 °C. Three fluoroscopic angles (RAO view 30°, RAO view 0°, LAO view 50°) were used to confirm the catheter position. The endpoint of the PV isolation was the creation of a bidirectional conduction block from the atrium to the pulmonary veins and vice versa. At least 20 min after a successful PV isolation, adenosine triphosphate was administered using intravenous isoproterenol (1–3 μg/kg/min) to provoke a reconnection of the PVs (dormant PV conduction). If any dormant PV conduction was observed, additional RF energy was applied at the earliest PV activation site until the dormant PV conduction was eliminated. If the AF was inducible after these procedures, sinus rhythm was restored by transthoracic cardioversion. During the procedure, bolus and additional heparin were administered to maintain an activated clotting time of 300–350 s. The blood pressure of the patient was continuously monitored, and SpO_2_, 12-lead electrocardiogram and ETCO_2_ measurements were taken during the ablation procedure. The duration of artificial respiration was defined as the total duration of the procedure. Total fluoroscopy duration, duration of radiofrequency energy, delivered radiofrequency energy, and total ablation points during the ablation procedure were calculated.

### Evaluation of the accuracy of the ETCO_2_ monitoring device

**Fig. 1:**
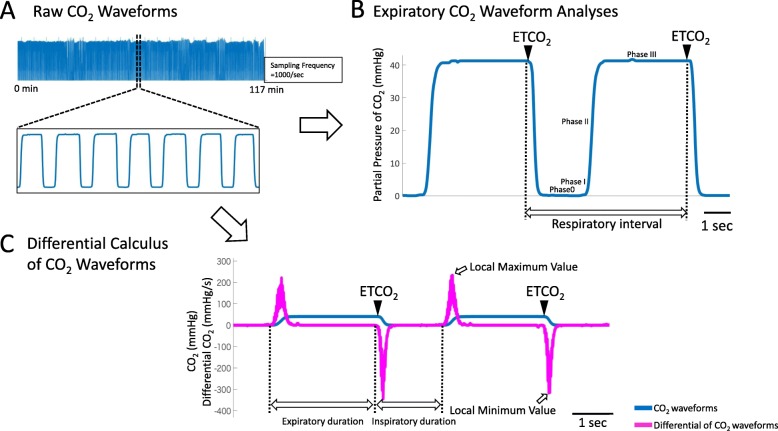
**a** and **b** Representative capnography waveforms obtained from an enrolled patient in this study. The expiratory phase was divided into 0, I, II, and III phases. The respiratory interval is defined by the distance between adjacent points of end-tidal CO_2_ (ETCO_2_). The interval between adjacent ETCO_2_ values is defined as respiratory interval. **c**: Representative capnography waveforms (blue lines) and their differential CO_2_ curves (magenta lines) obtained from an enrolled patient in this study. The differential CO_2_ curve was constructed, and the local maximum and minimum values were calculated where the respiratory intervals, and expiratory and inspiratory durations could be easily identified and calculated

First, we analyzed the correlation between pCO_2_ obtained from blood gas tests and ETCO_2_ obtained from capnography in 9 patients subjected to blood gas tests during RFCA. A linear correlation was seen between the 2 tests (Y = 0.8531*X + 4.888, R^2^ = 0.8808, *p* value = 0.0002). As a strong correlation was seen between the 2 tests, it is confirmed that measuring ETCO_2_ levels is appropriate for evaluating blood levels of carbon dioxide. Figure 1a, b shows the capnography waveforms obtained during measuring CO_2_. Generally, expiratory phase is from the point when the capnography starts increasing to the point that is defined as ETCO_2_, while the inspiratory phase lasts from the point that is defined as ETCO_2_ to the start of the expiratory phase. The expiratory phase was further divided into 0, I, II, and III phases (Fig. 1b). Next, the differential CO_2_ waveforms was constructed (Fig. 1c, magenta lines), and the local maximum values (maximum values) and local minimum values (minimum values) were calculated, where the respiratory intervals, expiratory durations and inspiratory could be easily identified and calculated. In this study, the analysis of respiratory parameters was performed as shown in Fig. 1c. A gas monitor (OLG-3800™; Nihon Kohden Co. Ltd., Japan) was used to monitor CO_2_.

### Data acquisition and analyses

**Fig. 2:**
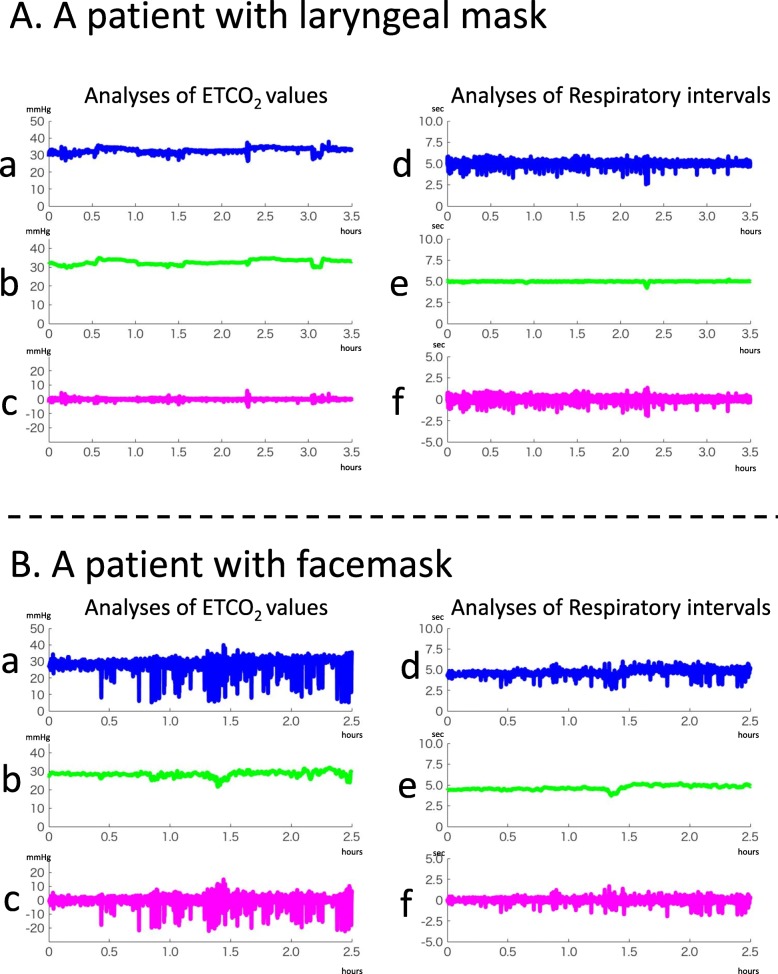
Representative results of chronological analyses of end-tidal CO_2_ (ETCO_2_) and respiratory intervals in a patient using a laryngeal mask. **A**. A patients with laryngeal mask. a and d: chronological changes of ETCO_2_ and respiratory intervals. b and e: moving averages of ETCO_2_ and respiratory intervals. c and f: chronological changes of standard deviations of ETCO_2_ and respiratory intervals. **B**. A patients with facemask. Representative results of chronological analyses of end-tidal CO_2_ (ETCO_2_) and respiratory intervals in a patient using a facemask. a and d: chronological changes of ETCO_2_ and respiratory intervals. b and e: moving averages of ETCO_2_ and respiratory intervals. c and f: chronological changes of standard deviations of ETCO_2_ and respiratory intervals

**Fig. 3:**
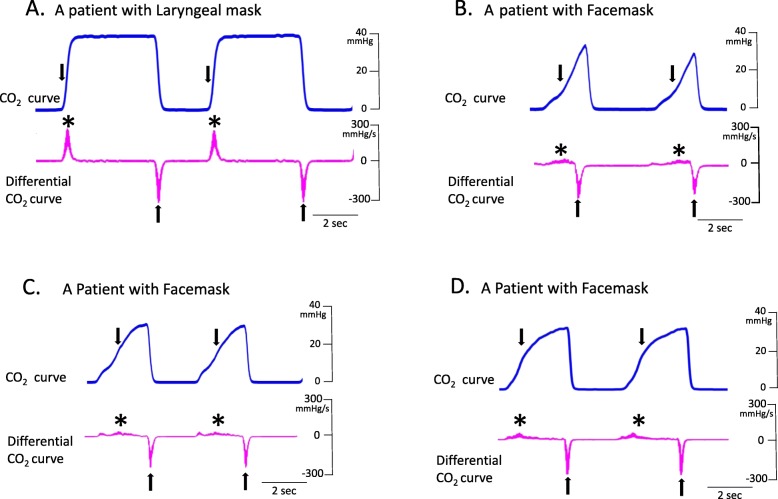
The representative data of the differential CO_2_ curves (bottom; magenta lines) from the capnography waveforms (top; blue lines). This was a respiratory waveform of the spontaneous respiration, recorded immediately before artificial ventilation management was initiated. **a**. Representative data in a patient using a laryngeal mask. **b**, **c**, **d**. Representative data in a patient using a facemask

After the patient was completely sedated, assisted respiration (using a mechanical ventilator) and continuous recording of CO_2_ (using an expiratory CO_2_ gas monitor) were started. The CO_2_ data from the expiratory gas monitor were transferred to a biological signal recorder (PowerLab 26T™; AD Instruments, Colorado Springs, CO, USA), which consisted of an A/D computer and another computer installed with a signal acquisition/analysis software (Chart Pro 5™; AD Instruments). The sampling rate was set at 1 kHz and was recorded as matrix data. The matrix data per patient was 9.46 × 10^6^ ± 2.59 × 10^6^. The analyzed ETCO_2_ samples per patient were 1555.3 ± 671.6 points. In order to identify the temporal changes in ETCO_2_, the local ETCO_2_ peaks of the CO_2_ waveform during the RFCA was identified, and to eliminate noise, the frequency between the peaks was set to ≤0.16 Hz and peaks greater than 0.5 Hz were excluded. Only the peak data were extracted and arranged in chronological order (Fig. 2A-a, B-a), and the central moving average of 20 points was recorded (Fig. 2A-b, B-b). The difference in the moving average from the peak ETCO_2_ sequence was calculated and the changes in standard deviation over time were plotted (Fig. 2A-c, B-c) to calculate the mean standard deviation. The differential coefficient per 0.001 s was calculated from the matrix data obtained from the biological signal recording device and a graph of the differential coefficient was constructed by arranging it in chronological order (Fig. 3: magenta lines). The respiratory interval was calculated by the peak-peak interval on the ETCO_2_ values of the CO_2_ curve (Fig. 2A-d, B-d), and the central moving average of 20 points was calculated (Fig. 2A-e, B-e). By calculating the difference in the sequence of the moving average from the sequence of the respiratory interval, the standard deviation of the data in chronological order was calculated (Fig. 2A-f, B-f). The differential coefficient curve during RFCA was constructed and the mean of the maximum increasing velocity of CO_2_ partial pressure during expiration and its standard deviation were calculated by identifying the peak on the positive side of the graph. By constructing the central moving averages at 500–800 points on the differential coefficient curve, the data was smoothened. The smoothened differential coefficient was defined as the expiratory time (from the point, where differential coefficients changes from negative to positive, to the point, where changes from positive to negative), and all the respiratory intervals were analyzed by calculating the mean and standard deviation. The peak on the negative side of the differential coefficient curve was set as the maximum lowering velocity of CO_2_ partial pressure during inspiration (Fig. 3: magenta lines) and its mean and standard deviation were calculated. Similarly, the central moving average was calculated and the time for the differential coefficient to turn from negative to positive was set as the inspiratory time. The sum of the expiratory and inspiratory durations was defined as the respiratory interval. It was confirmed that the error between the respiratory interval, which calculated using the sum of the expiratory and using the ETCO_2_ peaks was ≤0.05 s.

### Statistical analyses

All data were represented as mean ± standard deviation or percentage. When comparing the 2 groups, normally distributed parameters were analyzed using the t-test, and parameters without a normal distribution were analyzed using the Wilcoxon test. A linear analysis was used to examine the correlation between the 2 parameters and was represented with a partial correlation coefficient, significance probability, and a 95% confidence interval (CI). A multivariate analysis was performed on parameters with a significance probability < 0.1, which were analyzed by univariate analysis. All statistical analysis was performed using the SPSS 19.0 J software for Windows. The significance level was set at < 0.05.

## Results

Table 1 presents the background of the subjects included in this study. There was no variable (age, sex ratio, body mass index, medical history, echocardiography data, laboratory data, types of atrial fibrillation, 3% obstructive desaturation index, and the dose of drugs regarding the intravenous anesthesia) that showed a significant difference between the facemask and the laryngeal mask groups. Additionally, the dose of dexmedtomidine hydrochloride and propofol were not different between two groups.

**Table 1:** Baseline characteristics of enrolled patients with catheter ablation

Variable	Facemask group (*n* = 10)	Laryngeal mask group (*n* = 14)	*p* value
Age, years	67.9 ± 9.8	66.7 ± 9.6	0.771
Male sex, n (%)	6 (60)	10 (71.4)	0.559
BMI, kg/m^2^	23.1 ± 2.4	24.0 ± 3.1	0.425
Hypertension, n (%)	4 (40)	7 (50)	0.697
Diabetes mellitus, n (%)	1 (10)	3 (21.4)	0.615
Dyslipidemia, n (%)	1 (10)	2 (14.3)	0.759
Echocardiography data			
LA diameter (mm)	40.2 ± 6.3	36.1 ± 6.2	0.131
Ejection fraction (%)	67.9 ± 12.3	68.0 ± 7.3	0.981
Laboratory data			
eGFR (ml/min/kg)	58.9 ± 13.2	58.8 ± 10.9	0.989
Ln BNP (pg/ml)	4.3 ± 1.2	4.1 ± 0.6	0.560
Ln C-reactive protein (mg/ml)	−2.7 ± 0.8	−2.3 ± 1.2	0.324
Hemoglobin (g/dl)	14.2 ± 1.1	14.2 ± 1.9	0.977
Types of atrial fibrillation			
Paroxysmal atrial fibrillation	6 (60)	8 (57.1)	0.889
Persistent atrial fibrillation	4 (40)	6 (42.9)	0.895
3% ODI (n/h)	18.1 ± 12.1	17.7 ± 14.7	0.958
Analyzed ETCO_2_ samples (points/a patient)	1722.3 ± 682.0	1435.9 ± 662.7	0.314
Dose of Dexmedetomidine Hydrochloride (μg/kg)	2.7 ± 0.5	2.3 ± 0.6	0.103
Dose of Propofol (mg/kg)	1.6 ± 0.6	1.9 ± 0.7	0.244

The values are reported as the mean ± standard deviation. *BMI* Body mass index, *LA* Left atrium, *eGFR* estimated gromerular filtration rate, *BNP* Brain natriuretic peptide, *ODI* Obstructive desaturation index, *ETCO*_*2*_ End-tidal CO_2_

**Fig. 4:**
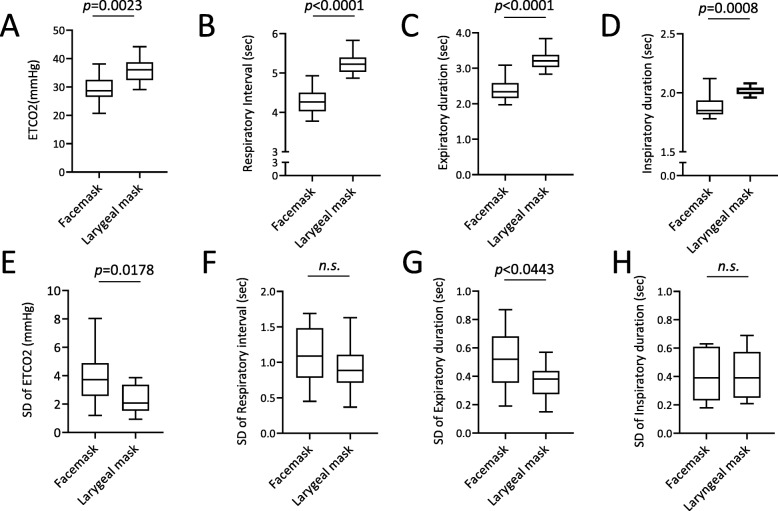
The respiratory parameters compared between the facemask and the laryngeal mask group during RFCA. **a**. Differences of mean end-tidal CO_2_ (ETCO_2_) values, **b**. Differences of mean respiratory intervals, **c**. Differences of mean expiratory duration, **d**. Differences of mean inspiratory duration, **e**. Differences of the standard deviation (SD) of ETCO_2_ values, **f**. Differences of the SD of respiratory intervals, **g**. Differences of the SD of expiratory duration, **h**. Differences of the SD of inspiratory duration

**Fig. 5:**
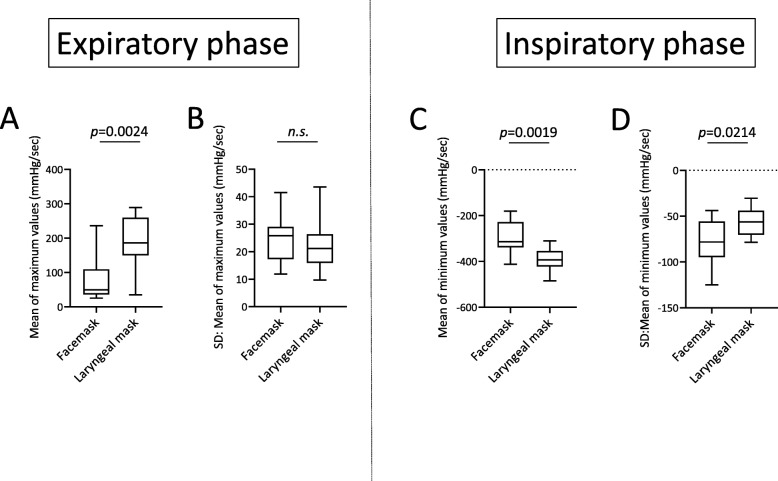
The maximum and minimum values and these standard deviations (SD), which were identified from the CO_2_ differential waveforms. **a**. Differences of mean of maximum values in the expiratory phase, **b**. Differences of the SD of maximum values in the expiratory phase, **c**. Differences of mean of minimum values in the inspiratory phase, **d**. Differences of the SD of minimum values in the inspiratory phase

**Fig. 6:**
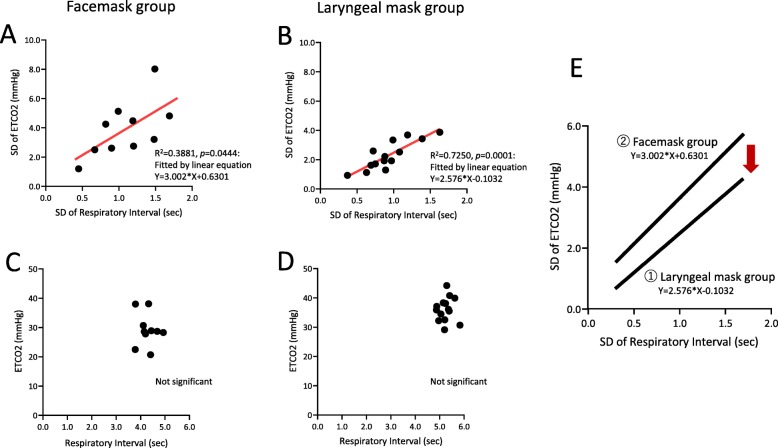
**a** and **b**: The respective plots of the facemask (**a**) and laryngeal mask groups (**b**) with the standard deviation (SD) of the respiratory interval set on the horizontal axis and that of end-tidal CO_2_ (ETCO_2_) values on the longitudinal axis. **e**. The difference of linear function of the facemask (**a**) and laryngeal mask groups (**b**) with the SD of the respiratory interval set on the horizontal axis and that of ETCO_2_ values on the longitudinal axis. **c** and **d**: The respective plots of the facemask (**c**) and laryngeal mask groups (**d**) with the respiratory interval set on the horizontal axis and the ETCO_2_ values on the longitudinal axis

**Fig. 7:**
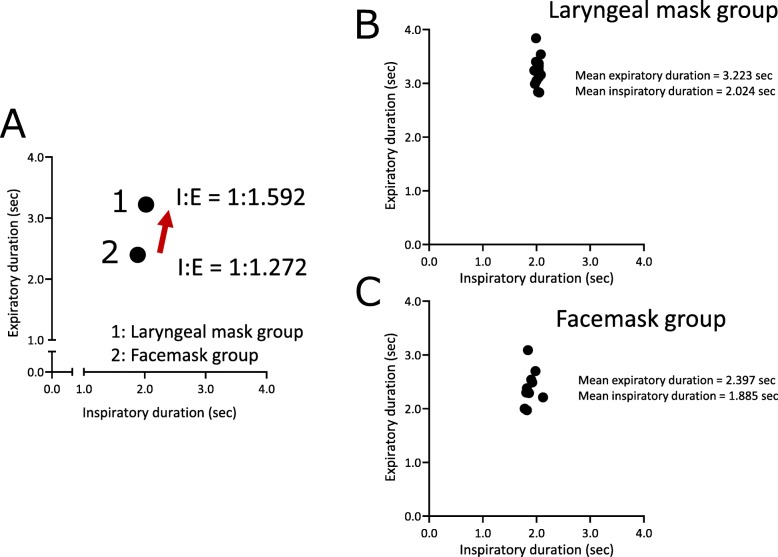
**a** The difference of mean inspiratory-expiratory (I/E) ratio between the laryngeal mask and facemask group. The relationship between expiratory and inspiratory duration in the laryngeal mask (**b**) and facemask group (**c**)

Figure 2 presents the representative data of the changes of ETCO_2_ values (Fig. 2A a-c) and that of respiratory interval (Fig. 2A d-f) in chronological order of the patients who used the laryngeal mask. Figure 2A-a shows the temporal changes in ETCO_2_, −b, the moving average, and -c, the difference (standard deviation: SD) between -a and -b. The moving average of ETCO_2_ progressed without any notable variation (mean ETCO_2_ = 32.5 mmHg) and variation per respiration was also relatively low (SD = 1.12 mmHg). Figure 2A-d presents the changes over time in respiratory interval, −e, the moving average, and -f, the standard deviation. As with the changes in ETCO_2,_ respiratory interval also progressed with no perioperative variation (SD = 0.63 s). Additionally, as shown in Fig. 2B, ETCO_2_ progressed at low values for patients used a facemask, (Fig. 2B-b; mean ETCO_2_ = 28.3 mmHg), and the variation in ETCO_2_ values was large (Fig. 2B-c; SD = 4.25 mmHg). Compared to the patients who used a laryngeal mask, the respiratory interval of the patients who used a facemask was mildly short (Fig. 2B-e; mean respiratory interval = 4.93 s), and the standard deviations were the same (Fig. 2B-f; SD = 0.82 s). Figure 3 shows the representative data of the differential CO_2_ curve (bottom; magenta lines) from the capnography waveforms (top; blue lines). This was a respiratory waveform of the spontaneous respiration recorded immediately before artificial ventilation management was initiated. In the CO_2_ curve (blue lines), phase II rises sharply in patients that used a laryngeal mask (Fig. 3a; down arrow), while in those that used a facemask, it rose gently (Fig. 3b-d; down arrow). Moreover, in patients that used a facemask, there were cases where phase II was convex at the top (Fig. 3b) and convex at the bottom (Fig. 3c and d). Analysis of the differential CO_2_ curve revealed that the peak of the velocity of rise of phase II in patients that used a laryngeal mask was high at approximately 300 mmHg/sec, while that of the patients that used a facemask was low at approximately 30 mmHg/sec (asterisks), respectively. The lowering velocity of the CO_2_ curve during inspiration was slightly higher in patients that used a laryngeal mask than that in those who used a facemask. Next, the respiratory parameters were compared between the 2 groups during RFCA (Fig. 4). The mean ETCO_2_ was significantly higher in the laryngeal mask group (36.1 vs. 29.2 mmHg, *p* = 0.0023, Fig. 4a) than that in the facemask group, and the SD was significantly lower in the laryngeal mask group (2.3 vs. 3.9 mmHg, *p* = 0.0178, Fig. 4e). The respiratory interval was significantly lower in patients who used a facemask than that in those who used a laryngeal mask (4.282 vs. 5.247 s, *p* < 0.0001, Fig. 4b). Additionally, there was no difference between the 2 groups regarding the SD for the respiratory interval (Fig. 4f). The expiratory duration was significantly longer (3.223 vs. 2.397 s, *p* < 0.0001, Fig. 4c) and the SD significantly shorter (0.361 vs. 0.513 s, *p* < 0.0443, Fig. 4g) in the laryngeal mask group than those in the facemask group. The inspiratory duration was significantly longer in the laryngeal mask group than that in the facemask group (2.024 vs. 1.885 s, *p* = 0.0008, Fig. 4d), and the SD for inspiratory duration showed no significant difference (Fig. 4h). The maximum values in the expiratory and the minimum values in the inspiratory phases were identified from the waveforms obtained from the CO_2_ differential waveforms (asterisks or arrows of magenta lines, Fig. 3), and the mean for these are shown in Fig. 5. In the expiratory phase, the maximum values of the laryngeal mask group were significantly higher than that of the facemask group (198.1 vs. 78.8 mmHg/sec, *p* = 0.0024, Fig. 5a); however, there was no difference in the standard deviations (Fig. 5b). Conversely, in the inspiratory phase, the minimum value in the laryngeal mask group was significantly higher than that in the facemask group (− 392.4 vs. -293.5 mmHg/sec, *p* = 0.0019, Fig. 5c). Furthermore, the SD of the laryngeal mask group was significantly lower (− 57.1 vs. -78.0 mmHg/sec, *p* = 0.0214, Fig. 5d). Figure 6a and b present the respective plots of the facemask and laryngeal mask groups with the SD of the respiratory interval set on the horizontal axis and that of ETCO_2_ on the longitudinal axis. In the laryngeal mask group, the SD of the respiratory interval and that of ETCO_2_ showed a strong positive correlation (*R*^2^ = 0.7252, *p* = 0.0001, Fig. 6b). Although a positive correlation was seen in the facemask group, there was more variation in the sample (*R*^2^ = 0.3881, *p* = 0.0444). Figure 6e shows the comparison of the slope obtained from the linear regression lines of Fig. 6a and b. The slope of the linear regression line was lower in the laryngeal mask group than in the facemask group. Additionally, there was no correlation between respiratory interval and ETCO_2_ for both groups (Fig. 6c and d). Figure 7 shows the relationship between expiratory and inspiratory durations for both groups. In the laryngeal mask group, the mean expiratory duration was 3.223 s, and mean inspiratory duration was 2.024 s (Fig. 7b). Additionally, in the facemask group, the mean expiratory duration was 2.397 s, and mean inspiratory duration was 1.885 s (Fig. 7c). The analysis of the mean of the inspiratory-expiratory (I/E) ratio showed that the ratio in the laryngeal mask group was 1:1.592 and that in the facemask group was 1:1.272, showing a prolonged expiratory duration in the laryngeal mask group (Fig. 7a). The use of a laryngeal mask resulted in a larger variation in the expiratory duration than that in the inspiratory duration. Table 2 shows the results of the RFCA, including total ablation procedures, the duration of the fluoroscopic procedure, total ablation points, and the delivered ablation energy. Multivariate analyses, including the use of a laryngeal mask, the ETCO_2_ value, and I/E ratio, showed that the ETCO_2_ value was the most influential parameter for the duration of the fluoroscopic procedure during the RFCA (Table 3).

**Table 2:** Ablation data in enrolled patients with catheter ablation

Variable	Facemask group (*n* = 10)	Laryngeal mask group (*n* = 14)	*p* value
Total duration of procedure, min	194.5 ± 29.1	162.5 ± 39.1	0.037
Total fluoroscopic duration, min	82.9 ± 17.6	55.9 ± 18.5	0.002
Duration of RF energy, min	40.3 ± 7.6	32.8 ± 9.0	0.042
Delivered RF energy, (×10^3^J)	73.5 ± 16.2	58.1 ± 16.1	0.030
Total ablation points, (points)	86.2 ± 14.0	64.6 ± 23.7	0.017

The values are reported as the mean ± standard deviation

*RF* Radiofrequency

**Table 3:** Uni- and multivariate analyses of influential factors on the fluoroscopic duration

Variable	Univariate analysis			Multivariate analysis		
	β	95% CI	*p* value	β	95% CI	*p* value
The use of laryngeal mask	−0.608	−42.55, −11.39	0.002			
ETCO_2_ value	−0.718	−3.954, −1.583	< 0.001	− 0.477	− 3.717, − 0.228	0.029
I/E ratio	−0.368	−78.30, 4.287	0.077			
Respiratory interval	−0.538	−35.50, −6.438	0.007			

*β* Partial correlation coefficient, *CI* confidence interval, *ETCO*_*2*_ end-tidal CO_2_;

The upper data are adjusted by age and gender

## Discussion

From this study, the following conclusions were drawn. 1) During the RFCA, the ETCO_2_ value of the laryngeal mask group was higher than that of the facemask group; the SD of the ETCO_2_ value was low. 2) The respiratory interval, expiratory duration, and inspiratory duration were significantly longer in the laryngeal mask group than those in the facemask group; the SD of the expiratory duration was significantly shorter. 3) In the expiratory phase, the mean value of the maximum increasing velocity of CO_2_ partial pressure was significantly higher when using a laryngeal mask than when using a facemask. The mean value of the maximum lowering velocity in the inspiratory phase was significantly high, and the SD was also high. 4) In both groups, a significant correlation was found between the SDs of the respiratory interval and ETCO_2_. The slope of the linear regression line was higher in the facemask group than in the laryngeal mask group. 5) The I/E ratio of the laryngeal mask group was significantly larger than that of the facemask group. 6) The ETCO_2_ value has the most influence on the fluoroscopy procedure duration during the RFCA.

The duration of an RFCA procedure is relatively long time to perform and is commonly performed under sedation with intravenous anesthesia to avoid pain caused by cauterization. However, during the procedure, respiration becomes unstable in terms of time and space, and stable respiratory management becomes necessary. Compared with tracheal intubation, airway management by a laryngeal mask has the advantages of lower invasiveness, easier insertion, and lower risk for injury to the pharynx and larynx [2]. Recently, various kinds of laryngeal masks could be used in the clinical practice according to patient’s peculiarity [8]. However, the use of laryngeal masks, compared with that of facemasks, has not been investigated extensively.

Sedation during the catheter ablation causes the upper airways to relax. This phenomenon, combined with gravity, causes some reactions: 1) the soft palate comes in close contact with the pharynx, resulting in impaired nasal breathing; 2) the base of the tongue drops causing obstruction in the upper airways; and 3) the epiglottis falls on the glottis obstructing the airways. The use of a laryngeal mask allows the airways to be secured even if there is obstruction due to the soft palate and sinking of the base of the tongue. Additionally, a laryngeal mask could prevent the obstruction of the airways by moving the epiglottis anteriorly [9]. When compared with a laryngeal mask, a facemask does not ensure direct patency of the airways; therefore, when a positive pressure is exerted by mechanical ventilation, adequately securing the pharyngeal part of the airways may be difficult in some cases [10]. In this study, both decreases in mean ETCO_2_ concentration and variations in ETCO_2_ concentration were shown in the facemask group (Fig. 4a and e). Additionally, both expiratory flow velocity and expiratory duration decreased in the facemask group as shown in Figs. 3, 4c, and 5a. This may have been a result of physical occlusion and stenosis of the upper airways, which caused expiratory instability. This study showed that variations (spatial variations) were seen in the amount of CO_2_ elimination due to stenosis of the airways. However, onset of variations (temporal variations) in the respiratory interval, which were frequently observed in patients with heart failure, were not experienced in patients with intravenous anesthesia (Fig. 4f).

As previously mentioned, inadequate expiratory ability and variation of the expiratory duration (Fig. 4g) could be caused by obstructions in the upper airways of patients with facemasks. Inadequate expulsion of CO_2_ due to obstructions in the upper airways could increase the CO_2_ concentration in blood and cause hyperventilation, which decreases the respiratory interval, expiratory duration, and inspiratory duration (Fig. 4b, c, d). Compared with the patients that used a facemask, those that used a laryngeal mask had a longer expiratory duration (2.397 s vs. 3.223 s) and a longer inspiratory duration (1.885 s vs. 2.024 s) (Fig. 7b and c). In both groups, the inspiratory duration could be secured at the minimum limit via the SIMV mode and pressure support of the mechanical ventilator. This resulted in a smaller decrease in the inspiratory duration than that in the expiratory duration (Fig. 4d). Additionally, mechanical ventilator support could not produce the SD of inspiratory duration in both groups (Fig. 4h). These results indicate that the laryngeal mask relieves obstructions in the upper airways, ensures adequate expiratory duration, adequate inspiratory duration, and prevents hyperventilation, resulting in respiratory stabilization (Fig. 4c) in patients under intravenous sedation.

The differential coefficients of the CO_2_ waveforms could be examined to evaluate the airflow velocities in the expiratory and inspiratory phases (Figs. 3 and 5). The capnography waveforms can clarify various abnormalities in respiratory conditions [11, 12]. For patients using laryngeal masks, the shape of the capnogram waveform per respiratory cycle resembled a square, while for those using facemasks, the shape resembled a triangle. The maximum value (maximum CO_2_ increasing velocity) of the CO_2_ concentration in the expiratory phase was distinctly higher in the laryngeal mask group, when compared with that of the facemask group, indicating that the use of laryngeal masks promotes patency of the airways and smooth expiration (Fig. 3: Asterisk and Fig. 5a). The minimal value (maximum CO_2_ lowering velocity) of the CO_2_ concentration in the inspiratory phase was also higher in the laryngeal mask group than in the facemask group, indicating that the inspiratory phase could also benefit from effective management of the upper airways (Fig. 5c). Additionally, the variations of the minimum value in the inspiratory phase were higher in the facemask group than in the laryngeal mask group. These variations were likely caused by the mechanical ventilator, which was set to a minimum of 10 air changes in the SIMV mode and a positive pressure supply of 6 cmH_2_O in the pressure support mode, and self-inspiration (Fig. 5d). The settings of the mechanical ventilation in the expiratory phase did not differ between two groups. Moreover, the ventilator setting did not change for each expiratory phase. These situations did not produce the differences in the variation of the maximum value in the expiratory phase between the two groups (Fig. 5b). The results of this study suggest that inadequate airway management decreased the maximum and minimum values of CO_2_ concentration, resulting in inadequate CO_2_ expulsion. These situations could lead to hyperventilation or decreased respiratory intervals during the RFCA procedure.

As shown in Fig. 6a, b, and e, when the variation in the respiratory interval is taken from the horizontal axis and the variation in ETCO_2_ from the longitudinal axis, the correlation coefficient becomes 1 unit larger. This result implies that the variation in CO_2_ expulsion ability during expiration is larger than the variation in the respiratory rhythm under intravenous anesthesia. Moreover, inadequate airway management techniques are characterized by further intensification of changes in ETCO_2_ values more than the variations in the respiratory interval. Variation in respiratory rhythm is commonly seen in heart failure patients, and is caused by a delay in circulation and increased sensitivity towards the medullary hydrogen ion [13, 14]. There were only a few heart failure patients in this study. Thus even if the CO_2_ partial pressure increased, abnormal respiratory rhythm occurred relatively less. As shown in Fig. 6a, b and e, the use of a laryngeal mask could secure the airways, resulting in increased CO_2_ expulsion ability, thus reducing the slope of the correlation coefficient (Fig. 6e, red arrow). This ability of the laryngeal mask causes CO_2_ concentration in blood to decrease, resulting in stable respiration.

Figure 7 shows the differences in the expiration-inspiration balance between the two groups. The I/E ratio of the patients that used laryngeal masks was 1:1.592 whereas it was 1:1.272 for those that used facemasks. Additionally, the inspiratory duration was longer in the laryngeal mask group when compared with the facemask group. These results clarify that the stabilization of respiration during RFCA was caused by a longer expiratory duration rather than inspiratory duration (Fig. 7a, red arrow). Preserving stable respiration is important while performing the ablation procedure. An increase in respiratory rate and variations in respiratory depth could worsen the fixation of the catheter during ablation, resulting in uneven cauterization and longer procedure duration. As a result of inadequate respiratory management, the success rate of ablation may decrease or other complications and disadvantages may arise during the RFCA procedure. Actually, total procedure and fluoroscopic durations were longer, and total RF energy and ablation points were higher in the facemask group than in the laryngeal mask (Table 2). Moreover, the ETCO_2_ value was the strongest parameter for the fluoroscopic duration during the RFCA procedure (Table 3). Therefore, the establishment of a stable ablation procedure using some respiratory devices, such as laryngeal masks, is required.

The limitations of this study were: 1) this was not a randomized study, 2) the number of subjects were comparatively fewer and the study was performed in a single institution, and 3) the success rates in the acute and chronic phases were not evaluated. 4) some objective monitoring methodology, such as bispectral index, to evaluate the depth of anesthesia could not be used. A large-scale in-depth study involving the RFCA success rate needs to be performed to establish the efficacy of the laryngeal mask.

## Conclusion

The use of a laryngeal mask could stabilize respiration during intravenous anesthesia, which could improve the efficiency of RFCA.

## Data Availability

The datasets used and analyzed during the current study are available from the corresponding author on reasonable request.

## Abbreviations

AF: Atrial fibrillation
BNP: Brain natriuretic peptide
CI: Confidence interval
CRP: C-reactive proteins
eGFR: estimated glomerular filtration rate
ETCO_2_: End-tidal CO_2_
I/E ratio: Inspiratory/expiratory ratio
LA: Left atrium
PEEP: Positive end expiratory pressure
PV: Pulmonary vein
RFCA: Radiofrequency catheter ablation
SD: Standard deviation
